# Variables associated with oral health-related self-efficacy – results of a cross-sectional study

**DOI:** 10.1186/s12903-023-03656-x

**Published:** 2023-11-28

**Authors:** David Bantel, Witold X. Chmielewski, Elmar Brähler, Yve Stöbel-Richter, Markus Zenger, Hendrik Berth

**Affiliations:** 1 Carl Gustav Carus Faculty of Medicine, Division of Psychological and Social Medicine and Developmental Neuroscience, Research Group Applied Medical Psychology and Medical Sociology, Technische Universität Dresden, Fetscherstr. 74, 01307 Dresden, Germany; 2 Institute for Psychological Psychotherapy, University of Bochum, Bochum, Germany; 3 Department of Psychosomatic Medicine and Psychotherapy, University Medical Center of the Johannes Gutenberg-University, Mainz, Germany; 4 Department of Medical Psychology and Medical Sociology, University Hospital Leipzig, Leipzig, Germany; 5 Faculty of Managerial and Cultural Studies, University of Applied Sciences Zittau/Görlitz, Görlitz, Germany; 6 Department of Applied Human Studies, University of Applied Sciences Magdeburg-Stendal, Stendal, Germany

**Keywords:** Self-efficacy, Dental anxiety, Dental hygiene, Oral health, Quality of life

## Abstract

**Background:**

Oral health-related self-efficacy (OH-SE) is pivotal for oral health and is associated with other oral-health related variables, such as dental fear and anxiety (DF/A) and dental hygiene behaviors (DHB). This study attempts to analyze associations between OH-SE and oral healthrelated variables in a German population to extend previous research by analyzing whether OH-SE can be predicted by these variables, as this might contribute to the development of treatment interventions.

**Methods:**

OH-SE, DF/A, oral health-related quality of life (OHRQoL), self-perceived dental condition, satisfaction with general health, DHB, and socioeconomic status were assessed as a part of the Saxon Longitudinal Study in an adult sample (n = 309, 56.3% female, all Saxon secondary school 8th graders in 1987). The associations of OH-SE with these variables were examined by means of correlation, multiple linear regression analyses, and group comparisons. Significance (p), standardized regression coefficients (β), and effect size (Cohen’s d) were calculated.

**Results:**

The correlation analyses revealed increased OH-SE to be accompanied by low levels of DF/A, high levels of OHRQoL, high levels of self-perceived dental condition, increased satisfaction with general health and socioeconomic status (all r ≥ 0.142; all *p* ≤ 0.013). In the regression analysis, OH-SE was mainly predicted by self-perceived dental condition and satisfaction with general health (R^2^ = 0.157) as well as by daily frequency of toothbrushing, OHRQoL, and socioeconomic status on a trend-level basis. In the group comparisons OH-SE was lower in participants with moderate for manifest DF/A and higher in individuals with higher OHRQoL, better self-perceived dental condition, increased satisfaction with general health, increased daily frequency of toothbrushing, more dental appointments, and above-average socioeconomic status (trend level; all t ≥ 1.57; *p* ≤ 0.059).

**Conclusions:**

In this cross-sectional study, high levels of OH-SE were mainly predicted by general health as well as self-perceived dental condition. It was also associated with decreased DF/A, increased DHB, higher OHRQoL, and higher socioeconomic status. Future research should analyze these associations in longitudinal designs to address whether interventions focusing on adherence to good DHB improve (dental) health and thus OH-SE. This might be a promising approach, particularly in relation to the treatment of DF/A.

## Background

Oral health is essential for overall health and quality of life and is closely related to psychological, physical, social, and economic well-being [1–3]. Because of its profound impact on overall health and well-being, previous research has analyzed its relationship with psychosocial factors and other variables to better understand how oral health can be promoted [4–6]. One dimension that has proven to be important regarding oral health is perceived self-efficacy as originally defined by Albert Bandura [3, 7–10]. The self-efficacy theory focuses on a person’s belief that he or she can successfully accomplish a particular task, and how this belief influences the person’s behavior and actions [7, 8]. It strongly emphasizes the active role of the individual in their own behavior and has been successfully linked to a variety of health behavior changes, such as weight management, smoking cessation, and alcohol use [11, 12]. With regards to oral health specifically, OH-SE has been positively linked to frequency of toothbrushing in children [13]. In addition, it has been shown that OHRQoL was indirectly associated with oral health knowledge through both self-efficacy and DHB [14]. Furthermore, a negative correlation between OH-SE and DF/A has been reported [15]. This finding is particularly relevant considering the detrimental effects of DF/A on oral health [16–18], as it has been shown that DF/A leads to a notable reduction in DHB, which is assumed to contribute to a vicious cycle of aggravated oral health and increased DF/A [19–22]. Since strengthening perceived self-efficacy is a well-established mechanism for improving health and health-related behaviors [7, 8, 11, 23, 24] in a wide variety of diseases [12, 25, 26], improving OH-SE might also be a promising approach in the context of oral health. Recent research has also documented a protective effect of more pronounced OH-SE on oral health, suggesting that strengthening OH-SE might reduce oral health-related problems in the future [3, 13, 14, 27].

However, before thinking about possible OH-SE-based interventions in the oral health domain, broader knowledge of the variables that potentially predict OH-SE is needed. Following the literature regarding an association between DF/A and OH-SE [15], it could be assumed that treatment interventions for DF/A might be adequate to improve OH-SE as well. On the other hand, research linking interventions improving DHB with changes in self-efficacy scores post-intervention [28] would suggest that treatment options focusing on behavioral changes (e.g., increased frequency of toothbrushing) might be more suited to address OH-SE. Therefore, the principal purpose of this study was to investigate whether OH-SE can be predicted by various oral health-related variables that showed associations with OH-SE in previous research. Based on the aforementioned results, we decided to include DF/A, OHRQoL, self-perceived dental condition, satisfaction with general health, and DHB (annual dentist appointments, cancellation of dentist appointments due to DF/A, and frequency of toothbrushing). We also included socioeconomic status, since a person’s socioeconomic status has been found to influence self-efficacy [29, 30]. The results could contribute to the future development of interventions to improve OH-SE by identifying its strongest predictors.

To our knowledge, the present study is the first to investigate OH-SE in a German population and the first to examine and to fill the gap as to which factors contribute to the expression of OH-SE. Measuring OH-SE comes with some challenges, as related scales mostly address specific behaviors (e.g. toothbrushing, flossing) or aim at capturing this construct in specific populations [3, 13, 14, 27, 31]. Because these measurements seemed inappropriate for our population, we followed Bandura’s original definition [7] and developed a new question (see methods section) to capture OH-SE. The one-question approach was also in line with the criterium of economy, which was especially important since this research was part of the Saxon Longitudinal Study, a large scale-study not only focused on oral health, but mainly aimed at collecting sociodemographic data in an East German population [32, 33].

In summary, this study particularly aimed to analyze the following hypotheses:

- OH-SE is positively associated with general health, self-perceived dental condition, DHB, and socioeconomic status (monthly income), and negatively associated with DF/A.
- OH-SE can be partly predicted by the aforementioned variables.
- OH-SE is increased in individuals with high levels of general health, self-perceived dental condition, DHB, socioeconomic status, and low levels of DF/A.

## Materials and methods

### Study design and recruitment of participants

This cross-sectional questionnaire study is part of a larger research project: the Saxon Longitudinal Study (German: *Sächsische Längsschnittstudie*). The Saxon Longitudinal Study is a recognized longitudinal analysis originally started by the Central Institute for Youth Research of the former German Democratic Republic (GDR; 32,33). Since its beginning in 1987, the same group of participants has been asked to fill out a comprehensive collection of questionnaires annually. At the beginning of the Saxon Longitudinal Study (1st wave in 1987, N = 1407), most participants were 14-year-old students attending 8th grade at the state-run Polytechnic Secondary School (German: *Polytechnische Oberschule*). They belonged to 72 classes from 41 secondary schools randomly selected from the then GDR districts of Leipzig and Karl-Marx-Stadt (now Chemnitz). The gender distribution was approximately equal. Thus, this age-homogeneous sample is considered representative for the East German cohort from 1973 on. After completing secondary school and reaching what was initially the planned end of the study (3rd wave in 1989, N = 1281), 587 participants agreed to cooperate in further research. The data analyzed in this study is from the 31st wave, which took place in 2019/20. In the 31st wave, a total of 323 participants participated in the survey. Due to missing data, 14 participants were excluded from our analyses, leaving a final sample size of 309. The study documents including all questionnaires were sent to the participants by mail and returned the same way. This research was conducted following the STROBE guidelines [34].

### Questionnaires

#### Dental anxiety scale (DAS)

DF/A was measured using the German version [35] of the internationally recognized Dental Anxiety Scale (DAS; 36). The self-assessment questionnaire is an economic alternative to other widely used instruments, for example the IDAF-4 C+ [21] or the MDAS [37]. It consists of four items and requires participants to rate their fear level by selecting the most adequate answer to each question (e.g. “When you are in the dentist’s chair waiting while the dentist gets the drill ready to begin working on your teeth, how do you feel?”). The responses to each of these questions are measured on a five-point scale ranging from 1 (relaxed) to 5 (highly anxious). The possible total score ranges from 4 to 20, with an average score of 8 in the general population and a defined cut-off score of 15, allowing the participants to be categorized into dental anxiety, moderate fear, and little to no fear (scores ≥ 15, 14 − 13, ≤ 12; [38]). The validity of the DAS has been demonstrated in several studies [38–41]. The reliability was rtt = 0.86 [36]. Cronbach’s alpha in this study was α = 0.93.

#### Oral health impact profile (OHIP-G5)

Oral health-related quality of life (OHRQoL) was measured with the German 5-item version [42] of the Oral Health Impact Profile (OHIP; [43]). The OHIP-5G includes the four dimensions “Oral Function”, “Orofacial Pain”, “Orofacial Appearance,” and “Psychosocial Impact”. It asks participants to select the most appropriate answer to each question (e.g. “Have you had difficulty carrying out your usual tasks because of problems with your teeth, mouth, or dentures?”). Each response was measured on a five-point scale ranging from 1 (very often) to 5 (never). For clarity, we decided to invert all scores, meaning that a higher total score would correspond to a higher OHRQoL. The OHIP-5G was developed to capture the maximum amount of information with as few items as possible. It proved to be a psychometrically sound and economic alternative to more extensive instruments and is considered a practical instrument for research and dental practice [44]. Cronbach’s alpha in this study was α = 0.81.

#### Dental hygiene behaviors (DHB) and other health variables

DHB was assessed using a total of three self-developed items. Participants were asked the following questions: 1) “How often do you go to the dentist each year?”, response options: never, once, twice, more often. 2) “Have you ever canceled or missed a dental appointment due to dental fear/anxiety?“, response options: yes, no. 3) “How often a day do you brush your teeth?”, response options: not at all, once, twice, three times, four times, more often. Participants’ general satisfaction with their general health was measured with one item (“When it comes to my present state of health, I am…”) and rated on a four-point response scale ranging from 1 (satisfied) to 4 (dissatisfied). Participants’ self-perceived dental condition was also measured with one item (“When you think about your teeth, what is their condition?”) and rated on a five-point response scale ranging from 1 (bad) to 5 (very good).

#### Oral-health-related self-efficacy (OH-SE)

Following Bandura’s original definition, OH-SE was measured with a self-developed item (“How much can you do yourself to maintain or improve the health of your teeth?”). Participants’ answers were measured on a five-point scale ranging from 1 (nothing at all) to 5 (a great deal). For clarity, all scores have been inverted, with a higher total score now corresponding to greater OH-SE.

#### Socioeconomic status

Participants’ socioeconomic status was assessed using one item (“What is your current personal net income in euros per month?”). Responses were measured on a 12-point scale asking for the personal monthly net income in EUR 500 increments (range: “I currently have no income” to “5,000 or more”).

### Statistical analysis

All statistical analyses were conducted using SPSS 28 (IBM). For descriptive statistics, mean (M) and standard error of mean (SEM) are reported.

To elucidate whether OH-SE relates to DF/A, OHRQOL, satisfaction with general health, DHB, and socioeconomic status, we analyzed for potential correlations between these variables. Pearson’s correlations were used for correlation analyses (small < 0.3, moderate < 0.5, and large effect ≥ 0.5), with statistical significance defined as p-value < 0.05.

A multiple linear regression analysis with stepwise inclusion with OH-SE as the dependent variable was used to examine whether OH-SE can be predicted based on the variables described above. These predictors were selected based on previous literature showing OH-SE to be related to poor self-rated oral health, general health, OHRQoL, DHB, socioeconomic status and DF/A [3, 13–15, 27]. Sex was included in the first step as a dummy variable to control for the effects of biological sex. In the subsequent step, all variables that significantly correlated with OH-SE (DF/A, OHRQoL, self-perceived dental condition, satisfaction with general health, cancellation of dentist appointments, frequency of toothbrushing) were included stepwise based on their correlation strength from the strongest to weakest predictor if additional variance was explained in the dependent variable. If predictors did not add predictive power to the regression, they were deleted in further analyses. In the last step, socioeconomic status as measured by personal monthly net income was added. The inclusion criterion was set to *p* = 0.05 and the exclusion criterion to *p* = 0.10. We reported standardized regression coefficients (β) and p-values.

To follow up on how the above variables affect OH-SE, we split each variable into two groups. This approach allowed us to classify groups according to established cut-off scores (DF/A) and to gain additional information based on dentist recommendations [45, 46] with regard to the frequency of toothbrushing per day and the number of annual dentist appointments, their subjective health assessments (satisfaction with general health, self-perceived dental condition, OHRQoL), cancellation of dentist appointments due to DF/A, and above-average socioeconomic status in order to further analyze contributors to OH-SE. In the DF/A group, participants with at least moderate DF/A were compared with participants with low or without DF/A. OHRQoL was split into two groups based on the median. Frequency of toothbrushing was split based on dentist recommendations of at least two times a day and three times a day for those exceeding expectations, dentist appointments according to a recommendation of at least twice a year, canceled dentist appointments based on whether any cancellation was present, and socioeconomic status based on a monthly net income of above EUR 2000, as indicated by the median split. The Shapiro-Wilk test was used to control for normal distribution of the data. In the case of normal distribution, independent t-tests were used to compare the two groups. Levene’s test was used to control for equal variance. Data that was not normally distributed was compared using Mann-Whitney U tests. Effect sizes were estimated using Cohen’s d (small < 0.2, medium < 0.5, and large effect ≥ 0.5). Statistical differences were considered significant with a p-value < 0.05.

The G*power 3 software program [47] was used to calculate an appropriate sample size for the desired statistical analyses. Based on a program-predefined effect size of f = 0.15, a significant level of *p* = 0.05 and power of 95% (1–ß = 0.95), a minimum sample size of N = 107 subjects was required for multiple linear regression analyses [48, 49]. The study was conducted in accordance with the Declaration of Helsinki [50]. The study protocol was approved by the Ethics Committee of the Technische Universität Dresden, Germany (EK EK8012011).

## Results

### Demographic characteristics

Of the 309 participants (mean age: 47.15 ± 0.03) included in the study, 56.3% were female. Average DAS and OHIP-G5 scores were 9.3 ± 0.22 and 23.22 ± 0.05 (median: 24), respectively. Concerning DF/A, 10.4% of participants classified for dental anxiety, 8.4% classified for moderate dental fear, and 81.2% had little to no DF/A. Regarding annual dentist appointments, 2.6% reported zero appointments, 43.8% reported one appointment, 46.0% two appointments, and 7.7% reported three or more appointments a year. A total of 18.2% reported cancellation of at least one annual appointment with their dentist due to self-proclaimed DF/A. Among the patients reporting cancellation of at least one annual dentist appointment, 32.1% qualified for moderate dental fear and 39.3% qualified for dental anxiety. Regarding the daily frequency of toothbrushing, 0.3% reported never brushing their teeth, 17.6% reported brushing their teeth once a day, 75.1% twice a day, 5.8% three times a day, 1.0% four times a day, and 0.3% more than four times a day. 13.1% of participants reported a very good self-perceived dental condition, while 43.8% reported a good, 30.8% a satisfactory, 9.9% a less good, and 2.9% a poor self-perceived dental condition. In terms of satisfaction with general health, 3.2% were satisfied, 23.9% were relatively satisfied, 46.0% were less satisfied than dissatisfied, and 23.9% were not satisfied. Regarding OH-SE, 37.1% reported that very much can be done by themselves to maintain or improve the condition of their teeth, 46.1% reported that a lot can be done, 15.3% reported that only a limited extend can be done, and 0.3% reported that very little to nothing can be done to maintain or improve the condition of their teeth. Concerning socioeconomic status, 0.3% of participants had no income, 1.3% below 500€, 3.9% below 1000€, 19.9% below 1500€, 22.9% below 2000€, 22.5% below 2500€, 12.7% below 3000€, 3.3% below 3500€, 2.9% below 4000€, 3.6% below 4500€, 2.3% below 5000€ and 4.2% above 5000€ net income per month.

### Correlation analyses

Small, significant correlations were found between OH-SE and DF/A (r = -0.178; *p* < 0.001) and OH-SE and OHRQoL (r = 0.244; *p* < 0.001). Moreover, a moderate, significant correlation between OH-SE and self-perceived dental condition was shown (r = 0.328; *p* < 0.001). Additionally, a small significant correlation was shown for OH-SE and satisfaction with general health (r = 0.225; *p* < 0.001). Regarding DHB, small trend-level significant correlations were found between OH-SE and cancellation of dentist appointments and between OH-SE and daily frequency of toothbrushing (all r ≥ 0.100; all *p* ≤ 0.079). Additionally, socioeconomic status showed a small significant correlation to OH-SE (r = 0.142, *p* = 0.013). All other correlations were not significant (all r ≤ 0.069, all *p* ≥ 0.225). All correlations are depicted in Table 1.

**Table 1:** Correlation Analyses based on OH-SE

	mean ± SEM / median of participants	1.	2.	3.	4.	5.	6.	7.	8.	9.
1. OH-SE	4.21 ± 0.040	1								
2. DF/A	9.30 ± 0.223	**-0.178** ^**^	1							
3. Socioeconomic status	median net income of 2000€	**0.142** ^*^	**-0.230** ^***^	1						
4. OHRQoL	23.22 ± 0.141	**0.244** ^***^	**–0.348** ^***^	**0.132** ^*^	1					
5. Self-perceived dental condition	3.55 ± 0.053	**0.328** ^***^	**0.275** ^***^	-0.052	**-0.484** ^***^	1				
6. Satisfaction with general health	2.04 ± 0.045	**0.225** ^***^	**0.273** ^***^	**0.197** ^***^	**0.112** ^*^	**-0.152** ^**^	1			
7. Annual dentist appointments	2.59 ± 0.038	–0.069	**–0.155** ^**^	-0.028	0.013	-0.085	0.003	1		
8. Cancelation of annual dentist appointments due to DF/A	1.82 ± 0.022	*–0.107* ^(*)^	**–0.511** ^***^	**0.194** ^***^	**0.255** ^***^	**-0.284** ^***^	**0.148** ^**^	0.012	1	
9. Daily frequency of teeth brushing	2.90 ± 0.031	*–0.100* ^*(*^ * ^*)*^	0.005	0.048	0.038	0.016	*-0.096* ^(*)^	**0.186** ^**^	0.019	1

Correlation analyses for oral health-related self-efficacy (OH-SE) and dental fear and anxiety measured with the DAS (DF/A), socioeconomic status as measured by net income, oral health-related quality of life measured with the OHIP-G5 (OHRQoL), self-perceived dental condition, satisfaction with general health, annual dentist appointments, cancellation of annual dentist appointments due to DF/A, and daily frequency of teeth brushing. Pearson’s r is reported. **Bold**: significant correlations, *italics*: trend-level significant correlations, ^***^p < 0.001; ^**^p < 0.01; ^*^p < 0.05; ^(*)^*p* < 0.10

### Regression analysis

To examine whether OH-SE can be predicted based on the variables described above, a regression analysis was conducted including the predictor sex in a first step. In the subsequent step, the predictors DF/A, OHRQoL, self-perceived dental condition, satisfaction with general health, dental appointments canceled due to DF/A, and daily frequency of toothbrushing were included. In the first step, the regression resulted in an overall R^2^ of < 0.001. The inclusion of the second-level predictors increased the R^2^ by 0.157 to an overall R^2^ of 0.157. However, only self-perceived dental condition (β = 0.196; *p* < 0.001) and satisfaction with general health (β = -0.108; *p* = 0.010) significantly predicted OH-SE, while daily frequency of toothbrushing contributed on a trend-level basis (β = 0.118; *p* = 0.098). When including socioeconomic status into the model the R^2^ increased to 0.170. Including this factor however only resulted in additional trend-level significant predictions by OHRQoL (β = 0.035; *p* = 0.093) and socioeconomic status (β = 0.035; *p* = 0.062). The regression analysis is depicted in Table 2.

**Table 2:** Regression Table for the Different Sequential Regression Models

Predictors of OH-SE	β^a^	SE^a^	BETA^a^	Sig. ^a^	R^2^	Sig	ΔR2
1 Sex	0.022	0.084	0.015	0.792	< 0.001	0.859	
**2 Self-perceived dental condition**	**0.196**	**0.048**	**0.259**	**< 0.001**			
**Satisfaction with general health**	**-0.135**	**0.050**	**-0.151**	**0.008**			
*OHRQoL*	*0.033*	*0.019*	*0.108*	*0.093*			
*Daily frequency of teeth brushing*	*0.118*	*0.071*	*0.091*	*0.098*			
Cancellation of annual dentist appointments due to DF/A	-0.094	0.116	-0.051	0.419			
DF/A	-0.008	0.012	-0.045	0.675	0.157	< 0.001	0.137
*3 Socioeconomic status*	*0.035*	*0.018*	*0.108*	*0.062*	*0.170*	*0.104*	*0.144*

Regression models for oral health-related self-efficacy (OH-SE). Model 1 only includes the predictor sex as a dummy variable. Model 2 includes the additional predictors for OH-SE which are: self-perceived dental condition, satisfaction with general health, daily frequency of teeth brushing, cancellation of annual dentist appointments due to DF/A, oral health-related quality of life measured with the OHIP-G5 (OHRQoL), dental fear and anxiety measured with the DAS (DF/A). Model 3 includes the additional factor socioeconomic status as measured by net income. Descriptive values for the regression with β (regression coefficient), SEM (standard error), BETA (standardized regression coefficient), R^2^ (proportion of explained variance), ΔR2 (change in R^2^); Sig (significance of the result to the left). **Bold**: significant group differences, *italics*: trend-level significant group differences

^a^ Coefficients are obtained from the final regression model

### Group comparisons

The group with dental anxiety and moderate dental fear (4.00 ± 0.098) showed significantly lower levels of OH-SE than the group with low to no dental fear (4.26 ± 0.04; t = − 2.58; *p* = 0.005; d = 0.375). Participants with higher levels of OHRQoL (OHIP-G5 median of 24 and higher) also showed higher levels of OH-SE (4.30 ± 0.05) than participants with lower levels of OHRQoL (4.02 ± 0.07; t = 2.64; *p* = 0.004; d = 0.307). The group that was dissatisfied with general health showed lower levels of OH-SE (4.02 ± 0.08) than the group that was satisfied with general health (4.28 ± 0.04; t = -2.919; *p* = 0.002; d = 0.373). Participants with lower levels of self-perceived dental condition showed lower levels of OH-SE (3.87 ± 0.13) than participants with higher levels of self-perceived dental condition (4.26 ± 0.04; t = -3.28 *p* < 0.001; d = 0.562). Participants with at least two annual dentist appointments scored higher on OH-SE (4.28 ± 0.05) than participants with fewer appointments (4.14 ± 0.06; t = 1.71 *p* = 0.044; d = 0.195). The group that canceled at least one dentist appointment due to fear scored lower on OH-SE (4.05 ± 0.09) than the group that never canceled a dentist appointment (4.25 ± 0.45; t = -1.88 *p* = 0.031; d = 0.278). Participants brushing their teeth at least twice a day did not differ from participants brushing their teeth less than two times a day (according to recommendations) in terms of OH-SE (4.23 ± 0.04; 4.16 ± 0.09; t = 0.618; *p* = 0.269; d = 0.091). However, participants exceeding recommendations by brushing their teeth at least three times a day had increased OH-SE in comparison to participants brushing less often (4.47 ± 0.11; 4.19 ± 0.04; t = 1.769; *p* = 0.039; d = 0.400). The group with an above-average socioeconomic status (net income above EUR 2,000) only scored higher on OH-SE on a trend-level (4.27 ± 0.06; 4.15 ± 0.06; t = -1.538 *p* = 0.059; d = 0.179). The group comparisons are depicted in Table 3.

**Table 3:** Group Comparisons based on Low and High OH-SE

Dental health variable	Group 1 OH-SE (mean ± SEM)	Group 2 OH-SE (mean ± SEM)	t-value	p-value	Cohen’s d
DF/A (moderate and manifest DF/A vs. without)	4.00 ± 0.10	4.26 ± 0.04	-2.58	0.005	0.375
OHRQoL (OHIP > 20 vs. OHIP < 20)	4.28 ± 0.40	3.73 ± 0.13	4.58	< 0.001	0.794
**Self-perceived dental condition (good vs. not so good)**	**4.26 ± 0.04**	**3.87 ± 0.19**	**3.28**	**< 0.001**	**0.562**
**Satisfaction with general health (satisfied vs. dissatisfied)**	**4.28 ± 0.04**	**4.02 ± 0.08**	**2.92**	**0.002**	**0.373**
**Annual dentist appointments (at least 2x vs. less than 2x)**	**4.27 ± 0.05**	**4.14 ± 0.06**	**1.70**	**0.044**	**0.195**
**Cancellation of annual dentist appointments due to DF/A (0 vs. more than 0)**	**4.24 ± 0.04**	**4.05 ± 0.09**	**1.95**	**0.027**	**0.278**
Daily frequency of teeth brushing (at least 2x vs. less)	4.23 ± 0.04	4.16 ± 0.09	0.62	0.269	0.091
**Daily frequency of teeth brushing (at least 3x vs. less)**	**4.47 ± 0.11**	**4.19 ± 0.04**	**1.77**	**0.039**	**0.400**
*Socioeconomic status (> 2000€ vs. < 2000€)*	*4.27 ± 0.06*	*4.15 ± 0.06*	*1.57*	*0.059*	*0.179*

Descriptive values for OH-SE group comparisons of dental fear and anxiety measured with the DAS (DF/A), socioeconomic status as measured by net income, oral health-related quality of life measured with OHIP-G5 (OHRQoL), self-perceived dental condition, satisfaction with general health, daily frequency of teeth brushing, annual dentist appointments, and cancelation of annual dentist appointments due to DF/A based on low and high OH-SE. **Bold**: significant group differences, *italics*: trend-level significant group differences

## Discussion

This study focused on the question of how OH-SE relates to DF/A, OHRQoL, DHB, self-perceived dental condition, satisfaction with general health, and socioeconomic status. To this end, the manifestation of these factors was surveyed in a questionnaire study and analyzed by means of correlations, regression models, and group comparisons.

In our study, approximately one-fifth of the sample qualified for manifest to moderate DF/A [51]. According to expectations, OH-SE was positively correlated with OHRQoL, self-perceived dental condition, satisfaction with general health, number of annual dentist appointments, and socioeconomic status, confirming that a more pronounced OH-SE is associated with greater quality of life, as well as overall health in general and oral health specifically [3, 14]. Additionally, significant negative correlations were found between OH-SE and DF/A, as well as OH-SE and the number of canceled dentist appointments due to DF/A (trend level), suggesting that increased OH-SE is accompanied by reduced DF/A and reduced avoidance behaviors. This is consistent with literature showing that higher levels of self-efficacy are associated with reduced psychopathological symptoms and better health [52–54]. Furthermore, a trend-level positive correlation was found between OH-SE and daily frequency of toothbrushing, which underlines the association between high OH-SE and increased health-related behaviors [7, 8, 11, 23, 24]. The regression analysis revealed OH-SE to be predicted by participants’ self-perceived dental condition, their level of satisfaction with their general health, as well as by their daily frequency of toothbrushing, their OHRQoL, and their socioeconomic status (trend level). This makes it possible to draw the following conclusions: Firstly, it supports the assumption that the concept of OH-SE might be closely related to, or be part of, a more general or broadly defined health-related self-efficacy [12, 55, 56]. More specifically, it suggests that individuals with high self-efficacy are more capable of altering their behavior to take better care of themselves, as evidenced by their health status, which is also reflected in increased economic success. Secondly, the regression advises that OH-SE is strongly dependent on the individual’s subjective measure of success, i.e., actual oral health and actual engagement in DHB. In other words, individuals seem to attribute their actual socioeconomic status and status of (dental) health stably and internally to their capability and effort to maintain such a good status quo [57].

Group comparisons demonstrated a stronger pattern of results. Here, participants with moderate to manifest DF/A or patients with canceled dentist appointments due to DF/A showed decreased OH-SE. In contrast, neither DF/A nor cancellation of dentist appointments predicted OH-SE in the regression, which suggests that this result might be biased due to the way OH-SE was assessed with the self-developed item “How much can you do yourself to maintain or improve the health of your teeth?” in the study. While participants with DF/A might still deem it theoretically possible to influence their dental health by adhering to beneficial DHB, they might be less successful at exercising good DHB due to their DF/A. In contrast to that and according to expectations, participants with higher levels of self-perceived dental condition, high OHRQoL, higher satisfaction with their general health, toothbrushing at least three times a day, and at least two annual dentist appointments or a net income of over EUR 2,000€ per month exhibited increased OH-SE. In combination with the results of the correlation and regression analyses, this pattern of results implies that advocating behavioral changes to adhere to good DHB and thus increasing dental health might be a feasible therapy approach to increase OH-SE. This approach is particularly promising as increased OH-SE has already been shown to increase DHB, decrease DF/A, and support the long-term improvement of dental health [13–15].

To our knowledge, this is the first study that analyzes which factors contribute to OH-SE. We consider this to be a major strength, as previous studies have examined OH-SE as a factor that influences oral health, DF/A, and OHRQoL without considering possible contributors to OH-SE itself. This might help to bridge an important gap in DF/A treatment. In addition, the large sample size allows us to make conclusions based on robust statistical analyses. The age homogeneity of the sample is also advantageous, as age effects have already been reported with respect to DF/A. Accordingly, specific findings should not have been averaged out by age distribution. Furthermore, it is one of few studies that examines a German sample, which might therefore allow us to generalize the results for European DF/A patients.

Some limitations must be considered as well. The follow-up question of whether DF/A predicts OH-SE if a more pathological concept of OH-SE [3] is applied, i.e., how much do you actually do for your dental health when under distress, still needs to be clarified. Additionally, to clarify this issue, a common definition of OH-SE is necessary. Furthermore, future therapy studies are required in which patients suffering from DF/A are treated with a focus on behavioral changes, with or without a focus on OH-SE, to further elucidate this matter. Future research should also examine a more heterogeneous sample in terms of age (particularly as DF/A has already been shown to decrease in elderly people [58]), education, and place of origin to allow for an increased generalization of our results. Additionally, future studies would also benefit from incorporating multilevel analysis to take the spatial clustering of individuals within specific areas into account [59]. Future studies should also consider multivariate analyses for group comparisons, as univariate findings might decrease the generalization of the results. Lastly, the association of socioeconomic context and general self-efficacy with OH-SE should be examined further to control for potential covariation in future studies.

In sum, our results illustrate that high levels of OH-SE are associated with reduced levels of DF/A as well as increased DHB, higher socioeconomic status, and an increased quality of life, both in terms of overall health and oral health specifically. The expression of OH-SE is mainly predicted by the subjective (dental) health status and engagement in DHB, which suggests that the individually assumed OH-SE is very strongly based on the outcome measure, i.e., self-perceived dental condition. As increased OH-SE is associated with improved DHB and oral health, this suggests that a behavioral therapy approach focusing on changes in adherence to good DHB should increase OH-SE, which could in turn promote oral health and reduce DF/A over the long term. This should be examined in future studies.

## Data Availability

The data presented in this study are available on request from the corresponding author. The data are not publicly available due to privacy issues.

## Abbreviations

DF/A: Dental fear and anxiety
OH-SE: Oral health-related self-efficacy
OHRQoL: Oral health-related quality of life
DHB: Dental hygiene behaviors
DAS: Dental Anxiety Scale
MDAS: Modified Dental Anxiety Scale
IDAF-4C: The Index of Dental Anxiety and Fear
OHIP: Oral Health Impact Profile
SPSS: Statistical Software for the Social Science
